# Supplementary material for: Variation of the Main Alkaloid Content in Equisetum palustre L. in the Light of Its Ontogeny
Source: Toxins (Basel). 2020 Nov 9;12(11):710. doi: 10.3390/toxins12110710 (PMC7696233; doi:10.3390/toxins12110710)
Supplement: Supplementary file 1 [file toxins-12-00710-s001.pdf]

Supplementary Materials: Variation of the Main Alkaloid Content in *Equisetum palustre* L. in the Light of Its Ontogeny

Jürgen Müller, Philipp Mario Puttich and Till Beuerle

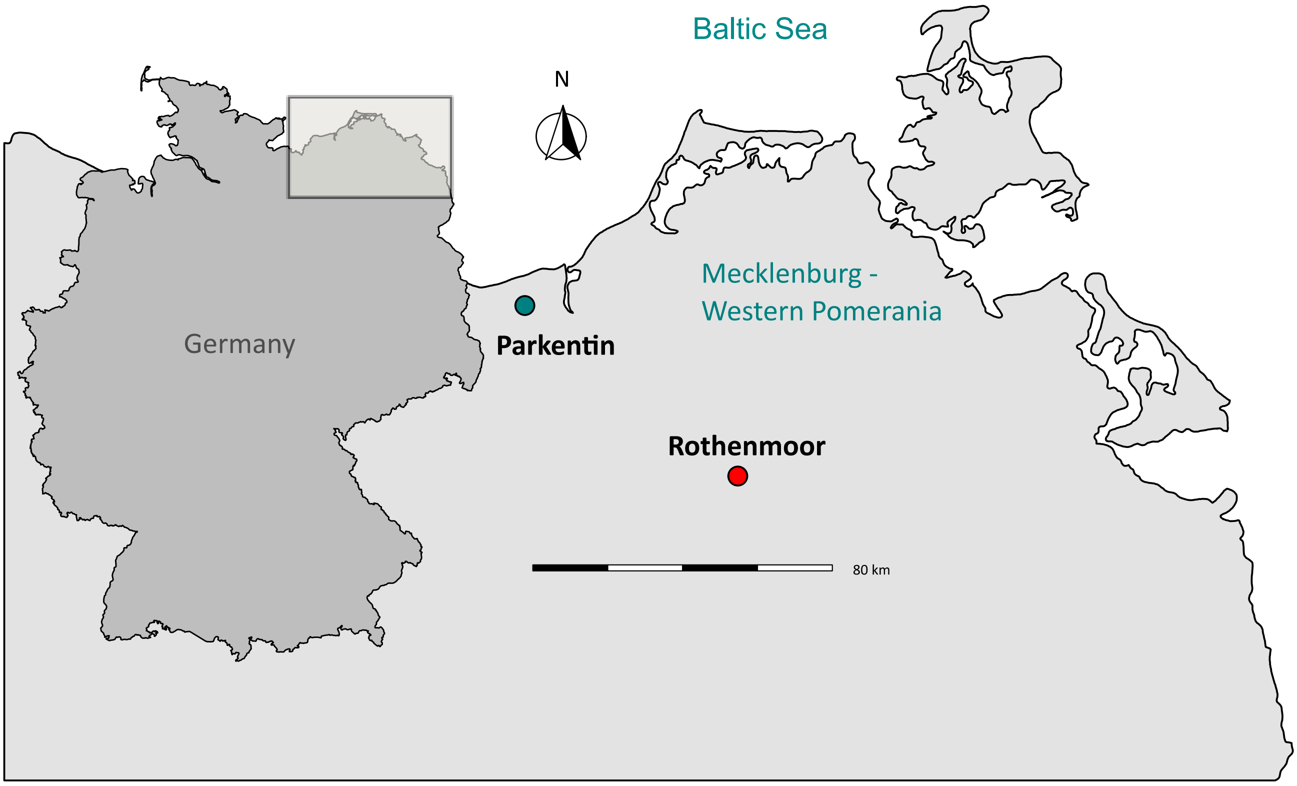

Figure S1. Location of the two sampling sites hosting *Equisetum palustre* - populations in northeast Germany.

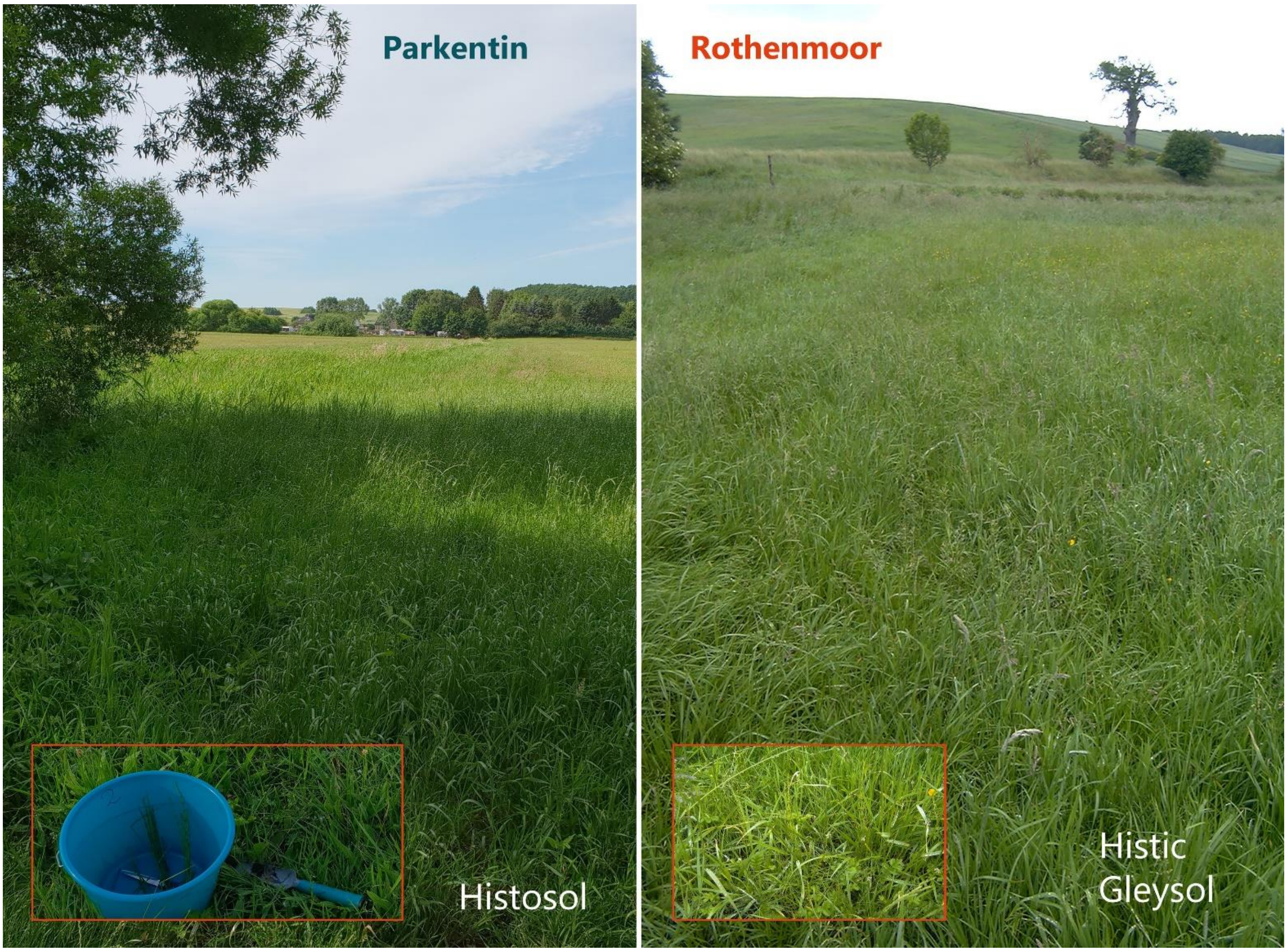

Figure S2. Impressions of the two sampling sites. Soil classification according to the World Reference Base for Soil Resources (WRB) is given in white letters.

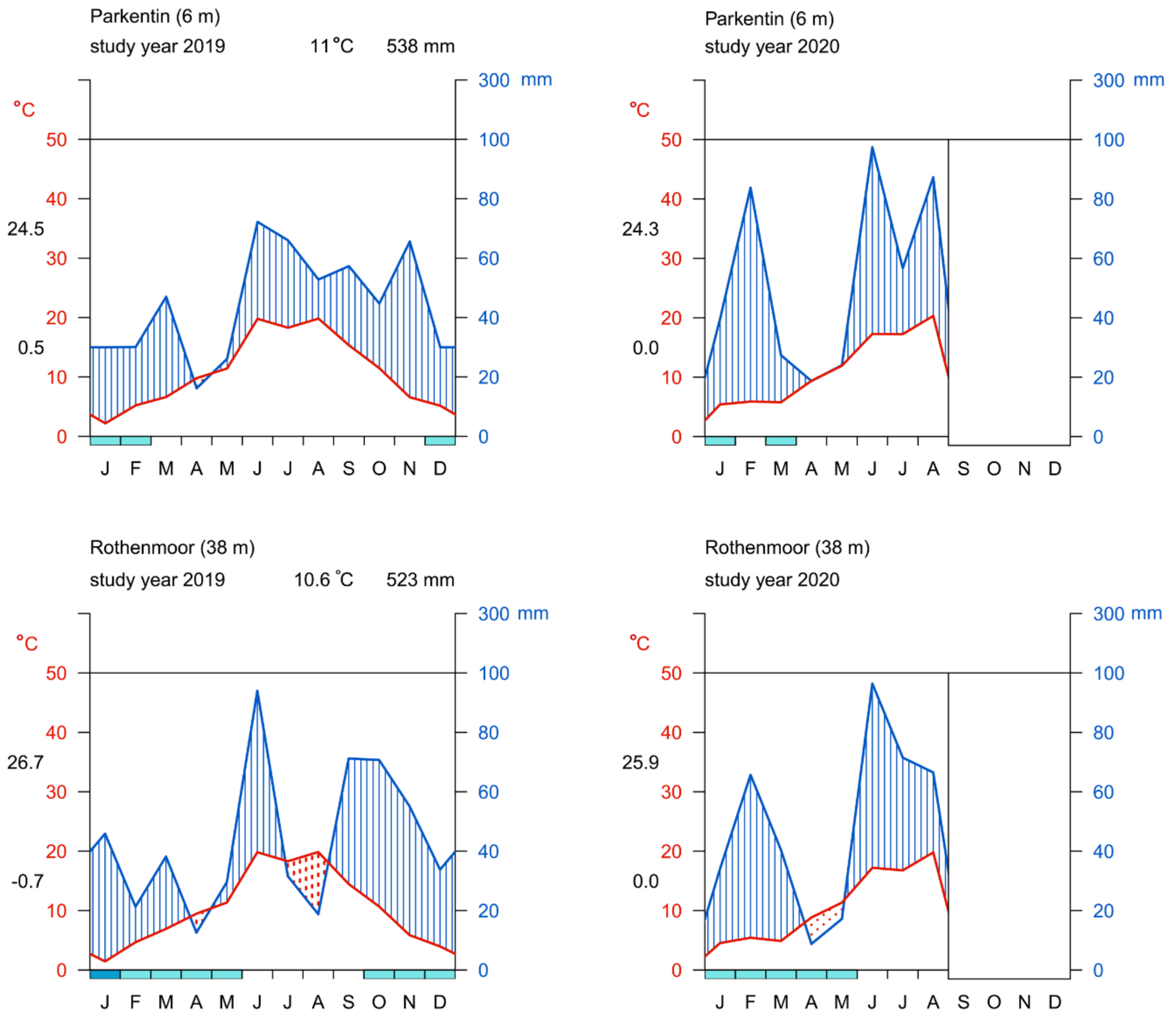

Figure S3. Climatological conditions during the sampling periods at the two sampling sites. Shown are Walter-Lieth climate diagrams of Warnemünde (weather station nearby the study site Parkentin) and of Teterow (weather station nearby the study site Rothenmoor) for the two main experimental years 2019 (top), and 2020 (bottom). The blue line represents the precipitation curve and the red line that of temperature. The blue vertical pattern depicts the humid periods, while the dotted red one shows when the aridity prevails. Values on the left axis are average maximum temperature of the warmest month and average minimum temperature of the coldest month. Frost likelihood is shown with flat rectangles just under the 0 °C axis. Upper right corner of the diagram shows annual average of temperature and annual total precipitation. We obtained the data from the German Weather Service DWD and constructed the diagrams using the R package climatol (Guijarro 2019).

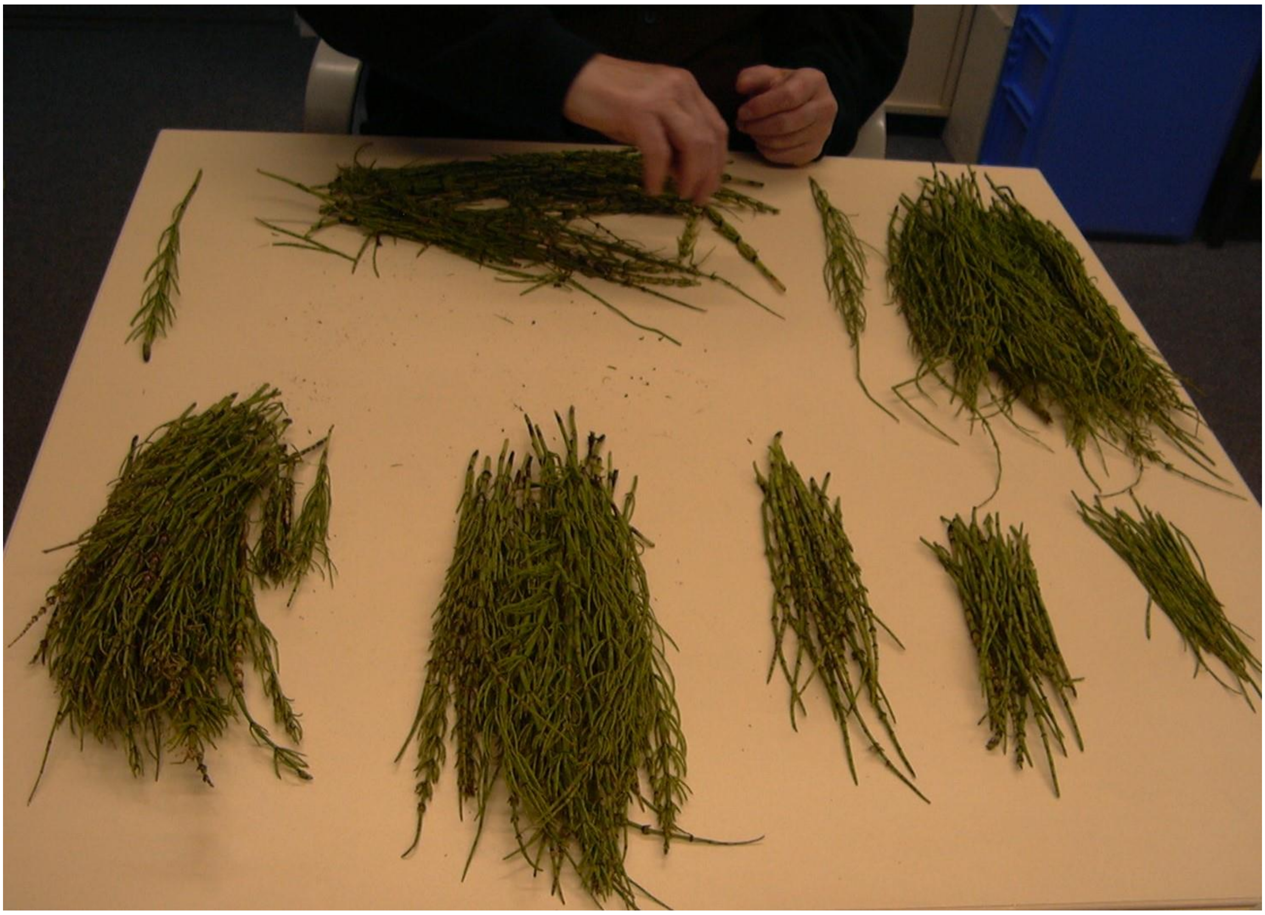

Figure S4. Dividing the horsetail collection into phenological stages immediately after field sampling. All measured alkaloid contents refer to such subsamples (cohorts).

Table S1. Summary of the applied ion transitions and compound specific MS/MS parameters for the detection of *Equisetum* alkaloids using HILIC-HPLC-ESIpos-MS/MS.

Analyte	Transitions [Da]*	DP [V]	CEP [V]	CE [eV]
Palustridiene	294.000 → 157.200	46.00	19.35	21.00
	294.000 → 114.200	41.00	19.35	38.00
	294.000 → 112.300	41.00	19.35	21.00
N^5-Formylpalustridiene	322.000 → 220.000	41.00	20.00	30.00
	322.000 → 199.000	61.00	20.00	27.00
	322.000 → 157.200	46.00	20.00	21.00
Alkaloid M 305	306.200 → 195.400	41.00	19.71	23.00
	306.200 → 152.000	36.00	19.71	37.00
	306.200 → 98.100	41.00	19.71	37.00
Alkaloid M 325	326.400 → 154.200	61.00	20.30	27.00
	326.400 → 96.100	41.00	20.30	42.00
	326.400 → 70.000	41.00	20.30	42.00
Palustrine	310.100 → 209.300	51.00	19.82	27.00
	310.100 → 154.200	61.00	19.82	27.00
	310.100 → 96.100	41.00	19.82	42.00
N^5-Formylpalustrine	338.100 → 251.000	51.00	20.64	23.00
	338.100 → 199.000	61.00	20.64	27.00
	338.100 → 156.100	66.00	20.64	27.00
N^5-Acetylpalustrine	352.200 → 265.400	56.00	21.05	31.00
	352.200 → 213.300	61.00	21.05	31.00
	352.200 → 100.100	46.00	21.05	47.00
Alkaloid M 323	324.100 → 213.100	41.00	20.23	27.00
	324.100→ 114.100	41.00	20.23	39.00
	324.100 → 70.000	41.00	20.23	35.00

*The respective quantifier ion transitions for each compound are marked in bold.

MS settings for all alkaloids: Curtain gas: 45 psi, ionization voltage: 5500 V, ion source temperature: 450 °C, ion source gas1: 35 psi and ion source gas2: 50 psi and collision activated dissociation (CAD) gas: high.

Table S2. Mixed effect model parameters for the response variable '*Equisetum*-type alkaloids' (sum of palustrine and palustridiene)

	Equisetum-type alkaloids			
Predictors	***Estimates***	***CI***	***p***	***df***
(Intercept)	630.97	23.48 – 1238.46	0.048	0.99
stage [1.5]	266.62	159.16 – 374.09	<0.001	141.56
stage [2]	−75.01	−118.49 – (−31.54)	0.001	140.08
stage [2.5]	−108.68	−167.50 – (−49.86)	<0.001	141.40
stage [3]	−237.91	−281.38 – (−194.43)	<0.001	140.08
stage [4]	−238.97	−304.80 – (−173.13)	<0.001	141.43
site [Rothenmoor]	−122.27	−183.77 – (−60.77)	<0.001	147.16
stage [1.5] * site [Rothenmoor]	−98.61	−221.78 – 24.56	0.116	141.27
stage [2] * site [Rothenmoor]	187.51	119.31 – 255.72	<0.001	140.40
stage [2.5] * site [Rothenmoor]	110.06	31.02 – 189.09	0.007	141.02
stage [3] * site [Rothenmoor]	174.57	108.68 – 240.46	<0.001	140.17
stage [4] * site [Rothenmoor]	172.66	88.14 – 257.19	<0.001	141.56
**Random Effects**				
σ^2	4544.69			
$\tau_{00\ GDD2}$	13884.69			
$\tau_{00\ year}$	0.00			
ICC	0.75			
N_{year}	2			
N_{GDD2}	11			
Observations	162			
Marginal R^2 / Conditional R^2	0.345/0.838			

Table S3. Results of the LMM-ANOVA with Satterthwaite's method to test the effects of ontogenetic stage, site, and the interaction of stage and site as fixed variables and the effects of year and GDD2 as random variables on the palustrine concentrations in shoots of marsh horsetail.

**Fixed Effect**	**NDF[1]**	**DDF[2]**	**F-value**	***P***
Ontogenetic stage	5	140.43	44.47	<0.001***
Sampling site	1	149.88	1.01	0.316 n.s.
Interaction stage × site	5	140.51	9.06	<0.001***
**Random Effect**	DF[3]	**Chi-square**		
Year of survey	1	0.00		n.s.
Growing degree days 2	1	142.16		<0.001 ***

[1] nominator Degrees of Freedom (NDF), [2] denominator Degrees of Freedom (DDF), n.s.-not significant; (***) *P*<0.001.

Table S4. Results of the LMM-ANOVA with Satterthwaite's method to test the effects of ontogenetic stage, site, and the interaction of stage and site as fixed variables and the effects of year and GDD2 as random variables on the palustridiene concentrations in shoots of marsh horsetail.

**Fixed Effect**	**NDF[1]**	**DDF[2]**	**F-value**	***P***
Ontogenetic stage	5	141.64	8.42	<0.001***
Sampling site	1	127.03	21.77	<0.001***
Interaction stage × site	5	141.71	2.26	0.052 n.s.
**Random Effect**	DF[3]	**Chi-square**		
Year of survey	1	1.18		0.277 n.s.
Growing degree days 2	1	57.00		<0.001 ***

[1] nominator Degrees of Freedom (NDF), [2] denominator Degrees of Freedom (DDF), n.s.-not significant (***) *P*<0.001.

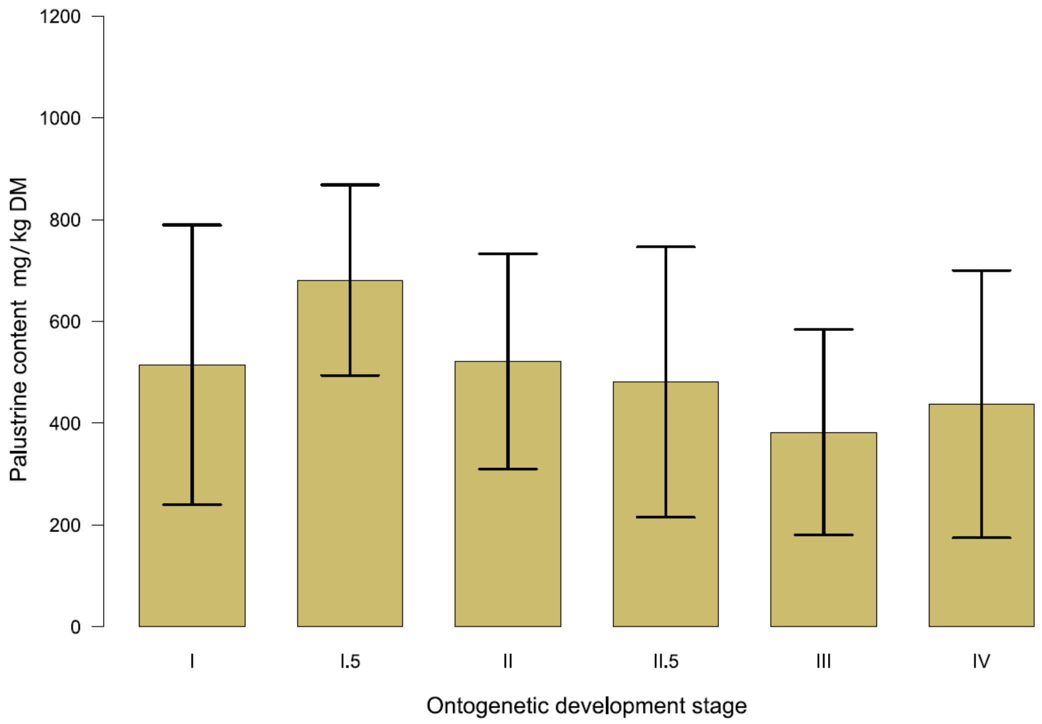

Figure S5. Concentrations of palustrine in the shoots of *E. palustre* L. in relation to the ontogenetic stage of plant development averaged over both study sites. Error bars = standard deviation of the means (± Sd).

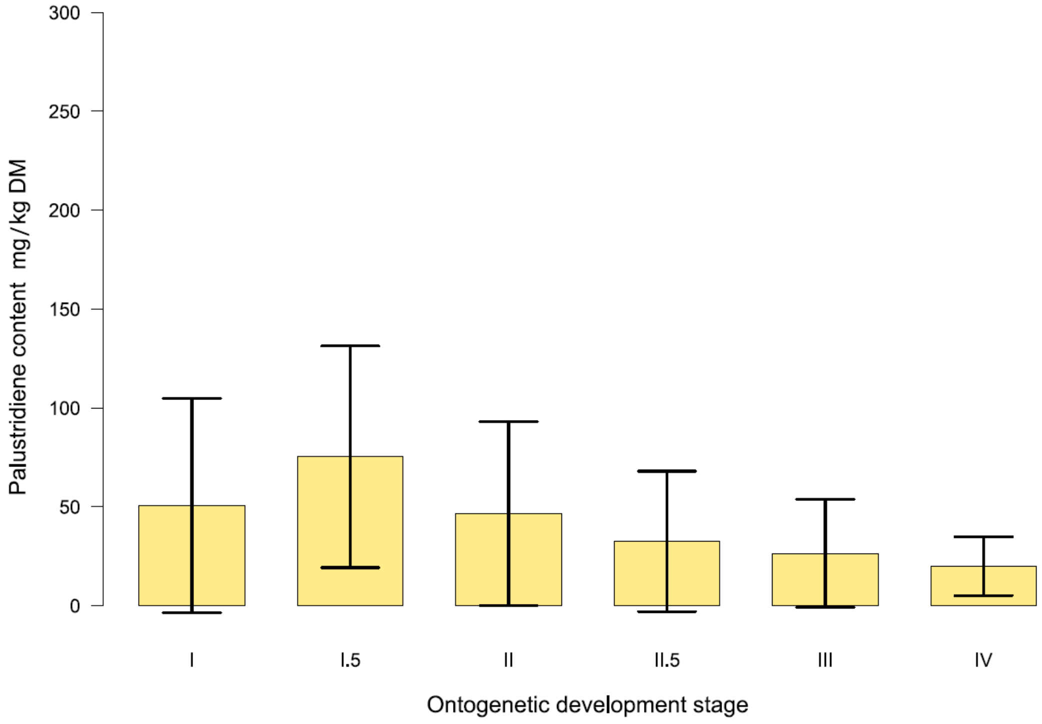

Figure S6. Concentrations of palustridiene in the shoots of *E. palustre* L. in relation to the ontogenetic stage of plant development averaged over both study sites. Error bars = standard deviation of the means (± Sd).

Note the differently scaled Y-axis when comparing Figure S6 with Figure S5.
